# Ferroptosis and Alzheimer’s disease: unraveling the molecular mechanisms and therapeutic opportunities

**DOI:** 10.3389/fcell.2026.1758041

**Published:** 2026-01-23

**Authors:** Yuan Fang, Zhongyu Han, Siming Yang, Juncheng Chen, Ruobing Li, Zhexu Zhang, Junhui Song, Danyan Wang, Yunqing Ban

**Affiliations:** 1 The Fifth Affiliated Hospital of Xinjiang Medical University, Xinjiang Medical University, Urumqi, China; 2 Institute of Nephrology, Zhongda Hospital, Southeast University, Nanjing, China; 3 Department of Neurosurgery, Zhujiang Hospital, Southern Medical University, Guangzhou, China; 4 The Affiliated Hospital of Qingdao Binhai University, Qingdao, China

**Keywords:** Alzheimer’s disease, ferroptosis, inhibitor, iron metabolism, mitochondrial

## Abstract

Ferroptosis is a novel form of regulated cell death. Compared with other types of cell death, it shows great differences in structure and biochemistry. This type of cell death is receiving increasing attention. For example, studies have found that it plays a key role in the development of neurodegenerative diseases underlying brain atrophy, such as Alzheimer’s disease (AD). AD is a chronic and worsening neurodegenerative disease. It poses a serious threat to the health and quality of life of the elderly. The pathology of AD is mainly the presence of extracellular beta-amyloid (Aβ) plaques and intracellular tau-based nerve fiber entanglement (NFTs). Although there are a large number of studies and interventions for AD, so far, no clinical drugs have been found that can stop the pathological progression of AD or cure it. Currently, treatment strategies for this disease only focus on alleviating clinical symptoms and do not achieve slowing disease progression or curing it. Ferroptosis is gradually considered to play a key role in the occurrence and development of AD. Research based on the AD model confirms that neuronal ferroptosis can be inhibited through pharmacology to reverse cognitive disorders. In this review, we first describe the key molecular mechanisms of ferroptosis, and then discuss how these mechanisms operate and develop in AD. Then, we give a detailed introduction to the latest treatments for AD, including iron chelators, antioxidants, and specific ferroptosis inhibitors. What is noteworthy is that this article emphasizes the analysis of the mechanisms of iron metabolism disorders, as well as the introduction of new drugs for the prevention, rather than the alleviation of AD.

## 1 Introduction

There are many causes of dementia, among which Alzheimer’s disease (AD) is the leading cause of neurological dementia. It presents an unrelenting erosion of mental agility, knowledge acquisition, and recall, coupled with newly surfacing behavioral and mood disturbances (Scheltens et al., 2021). Epidemiological data indicate that over 50 million individuals worldwide already live with AD. Patient numbers continue to surge, propelling AD toward the top tier of the globe’s costliest and deadliest diseases (Graff-Radford et al., 2021). Fresh statistics list Alzheimer-related illness as the nation’s fifth-leading killer. Hyperphosphorylated tau bundles into intracellular neurofibrillary tangles, igniting widespread nerve-cell death throughout the hippocampus and cortex. Meanwhile, shards of APP seed extracellular amyloid-β clumps that mature into senile plaques, marking the second signature lesion of the disease (Sengoku, 2020). Although toxic build-up of amyloid-β is considered the primary driver of Alzheimer’s pathology, clinical attempts to lower Aβ levels have so far failed to slow disease progression. Research reveals that the build-up of misfolded Aβ and tau starts silently one-and-a-half to two decades ahead of clinical symptoms appear (Long and Holtzman, 2019). Furthermore, the extent of this accumulation tightly parallels the dismantling of synapses and the demise of nerve cells in AD patients (Rajmohan and Reddy, 2017). However, the precise mechanisms that drive neurodegeneration in AD remain poorly understood.

In 2012, Dixon reported ferroptosis, which refers to a regulated, non-apoptotic form of cell death. It is characterized by iron-dependent oxidation of phospholipids within the cell membrane (Dixon et al., 2012). Ferroptosis is closely associated with iron homeostasis dysregulation, redox imbalance, and oxygen-dependent degradation of membrane lipids enriched in polyunsaturated fatty acids (PUFAs) (Jiang et al., 2021a). Numerous studies have shown that ferroptosis is triggered by the uncontrolled accumulation of lipid peroxides (Jiang et al., 2021a). Over the past decade, a growing body of evidence has increasingly implicated ferroptosis as a key culprit in a broad spectrum of diseases.

Ferroptosis has recently become a crucial research focus in neuroscience. A growing body of evidence links it to the onset and progression of various neurodegenerative diseases (NDs), including AD, Parkinson’s disease (PD), demyelinating multiple sclerosis (MS), as well as cerebrovascular disorders—whether acute ischemic stroke, nontraumatic intracerebral hemorrhage, or hemorrhage in the cerebral sulci. Emerging studies have revealed that with the continuous death of neurons in AD, the level of oxidative stress and the iron content in the brain are also steadily increasing (Lei et al., 2021). A growing number of ferroptosis protectants are being developed for AD, with natural products leading the way in fine-tuning this cell death pathway. Drugs approved by the U.S. Food and Drug Administration (FDA) for targeting AD pathology include iron chelators deferoxamine and deferiprone, as well as cholinesterase inhibitors galantamine and donepezil (Kim and Chung, 2021).

In this review, we systematically summarize ferroptosis and its common molecular mechanisms, as well as its specific role in the pathology of AD. Unlike other studies, we introduce the cellular microenvironment of ferroptosis and novel bioinformatics markers. Finally, we list current drugs targeting AD, aiming to identify new therapeutic targets for this condition.

## 2 Ferroptosis and its history

**FIGURE 1:**
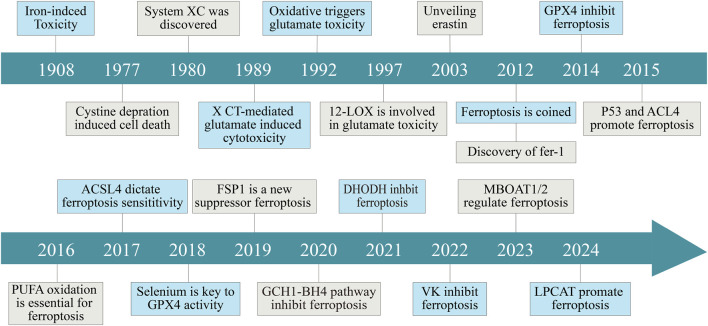
Historical timeline of key discoveries in ferroptosis research. Key events include the identification of System XC (1980), the discovery of erastin (2003), the coining of the term “ferroptosis” (2012), and the establishment of GPX4 as a central regulator. Subsequent research unveiled critical roles for lipid metabolism (e.g., ACSL4, PUFA oxidation), antioxidant systems (e.g., FSP1, DHODH, GCH1-BH4), and novel modulators such as vitamin K and LPCAT in ferroptosis regulation. This timeline highlights the evolving understanding of ferroptosis mechanisms and its relevance to disease pathophysiology.

Dixon and his colleagues first discovered ferroptosis in 2012 in cancer cells carrying mutations in the renin-angiotensin system (RAS), which is a type of abnormal suicidal cell fate regulated by iron-catalyzed reaction (Dixon et al., 2012) (Figure 1). Ferroptosis stands apart from other cell-death pathways in its biochemical signatures, genetic control, and structural appearance. Eagle’s pioneering work showed that simply stripping cysteine from the medium is enough to flip the death switch in cultured cells (Eagle, 1955). By contrast, enabling cells to produce cysteine internally confers robust resistance to this death pathway (Eagle et al., 1961). High-throughput screening revealed compounds that trigger selective demise of malignant populations carrying RAS driver mutations. From 2001 to 2003, Stockwell’s group used high-throughput screening to identify erastin, a novel agent that triggers apoptosis-independent cellular extinction exclusively within tumor cells driven by transforming RAS (Dolma et al., 2003).

This death pathway is driven by rising intracellular iron and oxidative pressure, yet it is readily halted by iron chelator (Yagoda et al., 2007). In 2008, Stockwell and colleagues extended their screening campaign and discovered two new synthetic molecules, RAS-selective death-3 small molecule (RSL3) and RAS-selective death-5 small molecule (RSL5). Both compounds selectively eliminated BJeLR cells through the same a lipid-peroxidation-driven, iron-requiring cellular sunset that shuns the apoptotic route (Yang and Stockwell, 2008). In parallel, Conrad’s group evidenced that GPX4 deletion triggers lipid-peroxide-dependent, programmed necrosis that is blocked by α-tocopherol (Seiler et al., 2008). Boosting levels of the glutamate-cystine exchanger SLC7A11 fortifies cells against this terminal track (Banjac et al., 2008).

Back in 2012, Stockwell’s lab revealed that erastin blocks cystine import through the XCˉ antiporter in HT-1080 fibrosarcoma cells. This triggers a ferric-catalyzed, caspase-shunning programmed necrosis program marked by way of PUFA-PL peroxide signaling buildup and by biochemical, morphologic, and genetic profiles unlike any previously described. Stockwell and colleagues proposed the concept of inhibiting ferroptosis by ferroptosis specific inhibitors (e.g., ferristatin-1 (Fer1)) (Dixon et al., 2012). In 2014, Yang et al. showed that deleting or over-expressing GPX4 respectively intensified or blocked the injury produced by ferroptosis-inducing compounds. Changing GPX4 levels did not protect cells from death stimuli acting outside the ferroptosis pathway, proving that GPX4 control is confined to ferroptosis alone. By genetically or pharmacologically manipulating GPX4, studies have firmly established its essential role in suppressing ferroptosis (Yang et al., 2014).

In 2016, Yang et al. demonstrated that PHKG2 governs lipid peroxidation by modulating iron supply to lipoxygenases. These enzymes exploit the delivered iron to peroxidize PUFAs specifically at bis-allylic carbons, thereby propagating the death cascade. Pre-incubating cells with deuterated PUFAs (D-PUFA) shielded these bonds from oxidation and consequently abolished ferroptosis. To sum up, ferroptosis is driven by the enzyme-catalyzed oxygenation of long-chain PUFAs (Yang et al., 2016).

Subsequently, Doll S discovered that ACSL4 sets the cell’s threshold for ferroptosis by sculpting its lipid profile (Doll et al., 2017). Strikingly, cellular populations simultaneously depleted of the glutathione peroxidase and the PUFA-activating transferase became highly unaffected by the ferroptosis, underscoring that ACSL4-controlled lipid changes are required for the death program to proceed. At the mechanistic level, the acyl-transferase ACSL4 fuels ferroptosis by loading membranes with elongated, highly unsaturated omega-6 lipids. Additionally, ACSL4 expression was markedly elevated in basal-like breast-tumor derivatives and served as a reliable predictor of their ferroptosis vulnerability. Moreover, Ingold’s 2018 work revealed that selenium is essential for warding off hydroperoxide-triggered ferroptosis. GPX4 incorporates selenocysteine to shield itself from irreversible over-oxidation, thereby maintaining its protective activity. In contrast, cells forced to express a cysteine-only GPX4 mutant lose this defense and become exquisitely sensitive to peroxide-triggered ferroptosis (Ingold et al., 2018).

Additionally, Doll S exploited cDNA library screens to fish out cancer-relevant loci from human tumor populations that can compensate for GPX4 loss. They identified the mitochondrial flavoprotein AIFM2 as a previously unknown suppressor of ferroptosis. AIFM2—now called FSP1—was first thought to promote apoptosis, but it actually protects cells from ferroptosis triggered by GPX4 loss. In summary, they identified FSP1 as a ferroptosis suppressor that operates independently of glutathione (Doll et al., 2019). Using a genome-wide activation screen, Kraft and VAN uncovered a set of ferroptosis-blocking genes headed by GTP cyclohydrolase1 (GCH1) and its downstream metabolites BH4/BH2(22). Consequently, GCH1 and its product tetrahydrobiopterin (BH4) suppress ferroptosis by reshaping the lipid landscape of cellular membrane (Kraft et al., 2020).

Moreover, Zou Y demonstrated that POR actively promotes ferroptosis in multiple lineages and cellular states, no matter which induction route is used. In other words, Cytochrome P450 oxidoreductase fuels ferroptosis by supplying the electrons that drive phospholipid peroxidation (Zou et al., 2020). Mao C and co-workers have recently identified DHODH as a mitochondrial ferroptosis defender, proposing its exploitation as a cancer therapeutic strategy (Mao et al., 2021). In 2022, Mishima E put forward the warfarin-resistant VK cycle acting as a robust ferroptosis brake (Mishima et al., 2022). At the same time, Zhang H L demonstrated that PKCβII phosphorylates ACSL4, thereby amplifying lipid peroxidation and driving ferroptosis.

Jung K’s 2023 study showed that melatonin plus zileuton boosts the AKT/MTOR/NRF2 pathway. This combination markedly eases ferroptosis-driven kidney injury and fibrosis, offering a promising therapeutic approach (Jung et al., 2023). Recently, research by Belaidi has confirmed that the PI3K/AKT signaling pathway can be activated by APOE. This signal inhibits the iron-catalyzed lipid peroxidation process by blocking the degradation of autophagic ferritin, that is, ferritinophagy (Belaidi et al., 2024).

## 3 The mechanisms of ferroptosis

### 3.1 Iron metabolism

**FIGURE 2:**
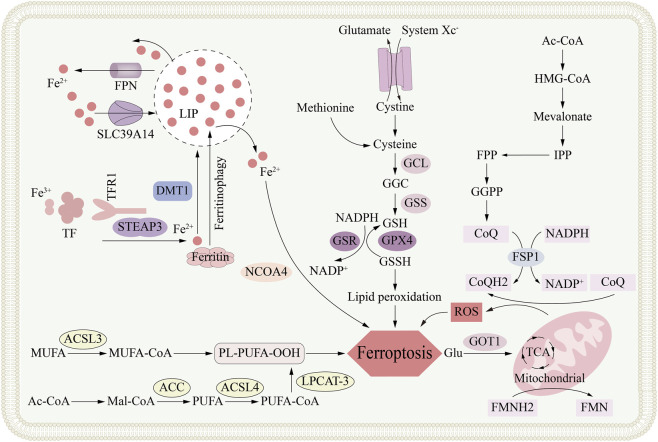
The metabolic mechanism of ferroptosis. Ferroptosis is an iron-dependent form of regulated cell death primarily driven by the accumulation of lipid peroxides. The core metabolic networks involved in ferroptosis, including cellular iron metabolism (via TFR1, STEAP3, DMT1, and ferritinophagy) which contributes to the labile iron pool (LIP); the glutathione (GSH) synthesis pathway (dependent on system Xc^−^ and enzymes GCL and GSS) that supports the antioxidant function of GPX4; the peroxidation of polyunsaturated fatty acids (PUFAs) esterified by ACSL4; and the compensatory FSP1-CoQ-NAD(P)H pathway that functions as an independent antioxidant system, collectively depicting the complex metabolic regulation of ferroptotic cell death.

The daily function of each neuron is entirely dependent on the metal iron. In neurons, it provides energy for mitochondrial respiration, drives neurotransmitter production, and promotes myelin formation (Ashraf et al., 2018; Wang and Pantopoulos, 2011). Now, dysregulation of iron metabolism is recognized as a key factor triggering ferroptosis. This metal-driven form of cell death has actually been known for decades (Jenkins et al., 2020). In organisms, iron exists primarily in two forms: ferrous iron (Fe^2+^) and ferric iron (Fe^3+^). Iron is typically stored and transported as ferric iron (Fe^3+^), and cells uptake iron through transferrin-dependent or transferrin-independent pathways (Johnsen et al., 2019). Transferrin and its receptor TFR are key partners in delivering iron to cells. Fe^3+^ binds to transferrin, and this complex is internalized into cells after binding to TFR1 on the cell surface, followed by endosome formation (Gammella et al., 2017; Koleini et al., 2021) (Figure 2).

A decrease in pH within the endosome causes Fe^3+^ to dissociate from transferrin; the subsequently released iron ions are reduced to the ferrous form by STEAP3, a six-transmembrane reductase selectively enriched in prostate epithelium (Koleini et al., 2021). Subsequently, after reduction, Fe^2+^ is imported into the labile iron pool (LIP) in the cytoplasm via DMT1 or its functional partner ZIP14 (Kühn, 2015). Simultaneously, The TF-TFR1 complex is recycled back to the plasma membrane, where it re-participates in subsequent cycles of iron uptake (Gao et al., 2015). Typically, metals are stored in ferritin within the cytoplasm; their export across the plasma membrane is entirely dependent on the mammalian transporter ferroportin-1 (FPN1) (Nemeth and Ganz, 2021). It maintains cellular iron homeostasis by transporting metals from the cytoplasm to the extracellular environment (Donovan et al., 2005). Study has discovered a new E3 ubiquitin ligase, RNF217, which regulates iron homeostasis by ubiquitinating its target, the iron transporter protein (FPN), and mediating its subsequent degradation (Jiang et al., 2021b). Research has found that, conversely, in a streptozotocin-induced type 1 diabetes rat model, reducing FPN levels leads to increased brain iron, causing ferroptosis, and ultimately resulting in cognitive impairment and decline (Hao et al., 2021). The repeated cycle of iron utilization, storage and external discharge collectively achieves the homeostasis of iron concentration in the body.

As mentioned before, DMT1 transports Fe^2+^ into the LIP. The accumulation of cytosolic free Fe^2+^, on one hand, initiates the apoptosis process driven by lipid peroxidation, known as ferroptosis, and on the other hand, may lead to iron homeostasis imbalance (Conrad and Pratt, 2019). Research has found that the Fenton reaction can be mediated by an excess of Fe^2+^ supplying electrons to hydrogen peroxide (H_2_O_2_) (Winterbourn, 1995). The Fenton reaction produces highly reactive oxygen species (ROS), such as hydroxyl radicals. These groups can damage membrane phospholipids through lipid peroxidation, and in severe cases, can induce ferroptosis (Xie et al., 2016). Superoxide dissociation can convert Fe^3+^ into Fe^2+^ through the Fenton-Habe-Wess cascading reaction catalyzed by iron. In addition, at the same time, the reaction produces superoxide anions that can trigger oxidative stress (·O_2_^−^) (Alayash, 2022).

An excess of cytoplasmic iron is fixed in the ferritin shell, which is composed of twelve light chains (FTL) and twelve heavy chains (FTH) peptides, forming a shell with 24 polymers that can accommodate about 4,500 iron atoms (Gál et al., 2008). IRP regulates the synthesis of ferritin according to iron levels. IRP1, IRP2, and iron-responsive elements (IREs) are jointly involved in this adjustment process. Its mechanism of action is to regulate iron metabolism protein-related genes after transcription: such as TFR1, DMT1, FTH and FPN1, so as to realize the iron uptake, storage, output and homeostasis of cell (Kühn, 2015; Bayeva et al., 2012). IRP1 and IRP2 can combine and act as specialized mRNA regulatory elements after activation by the LIP (Das et al., 2009).

The IRP-IRE interaction triggers a dual regulatory mechanism under low intracellular iron levels. For one thing, it increases cell iron uptake by promoting the synthesis of iron transporter DMT1 and transferrin receptor TFR1; for another thing, it inhibits iron discharge by inhibiting the translation of FPN1, which together increases the level of intracellular iron (Sun et al., 2022). In addition, ferritinophagy also participates in the regulation of intracellular iron levels, that is, it sends ferritin to lysosomes for degradation (Mancias et al., 2014). Through the degradation of ferritin, the stored iron is released. Studies have confirmed the key role of NCOA4 in ferritinophagy, acting as a regulatory factor that targets ferritin for degradation through the autophagy system (Fujimaki et al., 2019).

The peptide hormone hepcidin, released by the liver, governs body-wide iron balance. Its rising or falling levels direct ferroproteins internalization and degradation, thereby tightening or releasing the iron supply to match deficiency or overload (Murphy, 2009). When the body has too much iron, hepcidin production rises, boosting ferroproteins degradation to block further iron export and curb the overload. Conversely, during iron shortage, hepcidin output drops, allowing ferroproteins to stay on cell surfaces so more iron can enter circulation. Hepcidin lowers plasma iron by directing ferroportin-1 to lysosomal destruction in enterocytes, macrophages, and hepatocytes, thereby shutting down cellular iron export (Murphy, 2009). Furthermore, hepcidin expression is induced by an inflammatory/infectious state. Under inflammatory conditions, the IL-6–JAK2 axis is the principal route that switches on the hepcidin promoter (Chen et al., 2021a). Hepcidin activity is also restrained by negative regulators. Erythroferrone (ERFE) curbs its expression when hematopoiesis is impaired, whereas platelet-derived growth factor-BB (PDGF-BB) does so under hypoxic conditions (Gao et al., 2019; Zhang et al., 2024).

Disrupted iron balance and rising ROS levels drive lipid peroxidation that injures cellular integrity. A drop in GSH or loss of GPX4 activity is usually enough to trigger ferroptosis (Eagle, 1955). An iron overload in the LIP—or any upset in cellular iron balance—can ignite ferroptosis (Yan et al., 2021). A surge of iron in the LIP overloads the cell, fueling Fenton and Haber-Weiss chemistry that spawns free radicals and devastates proteins and lipids (Song et al., 2018). In the Fenton process, hydrogen peroxide converts Fe^2+^ to Fe^3+^ while spawning the extremely aggressive hydroxyl radical (OH^−^). Superoxidase reduce Fe^3+^ back to Fe^2+^ through the Haber-Weiss reaction, releasing superoxide anions (·O2−) and promoting oxidative stress damage (Alayash, 2022). This can lead to lipid peroxidation and eventually induce ferroptosis (Stockwell et al., 2017). Iron plays a key role in the process of driving the ferroptosis. On the contrary, researchers widely believe that NADPH-driven POR enzyme and NADH-powered CYB5R1 enzyme are catalysts for destroying cell membranes. Under the mediary of these enzymes, electrons are transferred to oxygen to produce H_2_O_2_ and then H_2_O_2_ through Fenton reaction oxide film phospholipids, which eventually leads to the production of ferroptosis (Yan et al., 2021). Unlike the production of other peroxides, the process of NADPH oxidase (NOX) causing ferroptosis the direct production of reactive oxygen (ROS) that leads to lipid oxidation damage (Alvarez et al., 2017).

### 3.2 Lipid peroxidation

The increase of lipid peroxidation (LPO) caused by lipid redox disorder is a key condition for ferroptosis. This lipid peroxidation can be produced through enzymatic reactions mediated by lipoxygenases (LOXs) or by non-enzymatic reactions mediated by Fenton (Lin et al., 2021). Lipid metabolism, especially lipids rich in double bonds, that is, the lipid metabolism of polyunsaturated fatty acids (PUFAs), is essential to induce ferroptosis. This is because polyunsaturated fatty acids contain easy-to-extract diallylated hydrogen, so these lipids are the main raw materials for peroxidation. In this process, carbon-centered lipid free radicals (PL•) will be generated; if lipid free radicals (PL•) meet O_2_, they will combine with them to form peroxide free radicals (PL-OO•), and these peroxide free radicals will extract hydrogen from neighboring polyunsaturated fatty acids (PUFAs) to produce lipids hydroperoxides (PL-OOH) (Conrad and Pratt, 2019). Then lipid peroxide (PL-OOH) decomposes to produce the products of oxidative damage proteins, which mainly include 4-hydroxyonenaldehyde (4-HNE), propylene glycol (MDA), *etc.* This amplified chain reaction eventually leads to damage to the cell membrane, which leads to the ferroptosis (Jiang et al., 2021a).

ACSL4 converts polyunsaturated fatty acids (PUFAs) into polyunsaturated fatty acid coenzymes A (PUFA-CoA) to activate them, and then, LPCAT3 esters these polyunsaturated fatty acid coenzymes A (PUFA-CoA) into phospholipids to produce polyunsaturated fatty acid phospholipid (PUFA-PL) substrates for peroxidation reaction (Zheng and Conrad, 2020). The lipoxygenase (LOX) enzyme group mainly includes 5-lipoxynase, 12-lipoxygenase and 15-lipoxygenase isoenzyme, which together form the lipoxygenase family. It should be noted that the family enzyme needs iron or related metal ions to catalyze its activation. These catalysts promote the oxidation of polyunsaturated fatty acids and damage the cell membrane. Through the discovery of the above process, we have deepened our understanding of the process of ferroptosis induced by lipid peroxidases (LOX) (Yang et al., 2016; Haeggström and Funk, 2011).

The study found that 15-lipoxynase (15-LOX) can selectively act on phospholipids (PUFA-PLs) containing polyunsaturated fatty acids and convert them into phospholipid-bound peroxides (PL-OOH), which is the key process of ferroptosis. Therefore, although high unsaturation can maintain the fluidity and functionality of cell membranes, polyunsaturated fatty acid phospholipids (PUFA-PLs) also make these membranes extremely susceptible to oxidative damage (Kagan et al., 2017). In contrast, monounsaturated fatty acids (MUFAs) are more able to resist peroxidation and avoid membrane damage (Magtanong et al., 2019). Therefore, research confirms that replacing polyunsaturated fatty acids (PUFAs) in the membrane with monounsaturated fatty acids can reduce the production of lipid peroxidation by providing single unsaturated fatty acids (MUFAs) to cells externally or increasing their endogenous synthesis. This fatty acid alternative reduces the sensitivity of cells to ferroptosis (Yang et al., 2016; Tesfay et al., 2019).

Zhang and his colleagues also discovered another new pathway that promotes ferroptosis, namely, the PKCβII-ACSL4 regulatory pathway (Zhang et al., 2022). PKCβII, an isoform of the protein kinase C family, is phosphorylated and gradually accumulates on the membrane when phospholipid peroxidation is exacerbated. This time- and dose-dependent response makes PKCβII a potential sensor for elevated lipid peroxide levels. Meanwhile, activated PKCβII promotes the assembly of the PKCβII–ACSL4 complex. Activated PKCβII phosphorylates ACSL4 at the Thr328 site, facilitating ACSL4 dimerization and increasing PL-PUFA levels, ultimately triggering ferroptosis (Zhang et al., 2022).

### 3.3 Mitochondrial metabolism

Mitochondria is not only an essential biological energy hub and a cell power plant that produces ATP, but also mediates the process of cell death (Zorov et al., 2014). Whether mitochondria directly drive ferroptosis is still controversial, but there is no doubt that it is the main source of reactive oxygen (ROS) in mammalian cells (Murphy, 2009). More and more evidence shows that mitochondria promote the initiation and development of ferroptosis through environmentally sensitive metabolic pathways (Chen et al., 2021a) A variety of mitochondrial metabolic pathways participate in the process of triggering ferroptosis.

Research shows that the metabolic process of glutamine, especially its hydrolysis process, is a key factor leading to ferroptosis (Gao et al., 2015). In addition, the mitochondrial tricarboxylic acid (TCA) cycle is also involved in the ferroptosis. Specifically, after entering the cell, glutamine decomposes and releases alpha-ketoglutaric acid under the action of glutaminase, and the latter enters the TCA cycle to provide energy for ferroptosis (Gao et al., 2015). At the same time, the tricarboxylic acid (TCA) cycle can promote the production of lipid peroxidation by producing reactive oxygen (ROS), adenosine triphosphate (ATP) or phospholipids (PUFA-PL) containing polyunsaturated fatty acids, thus causing ferroptosis (Gao et al., 2019). Mitochondria is the main source of intracellular reactive oxygen (ROS). The electron leakage of mitochondrial respiratory transfer chain complexes I and III leads to the generation of superoxide anions, which are later converted into hydrogen peroxide (H_2_O_2_) by superoxide dismutase (SODs) (Murphy, 2009). Peroxide promotes the Fenton reaction catalyzed by Fe^2+^, which further promotes the oxidative decomposition process of polyunsaturated phospholipids.

In addition, mitochondrial-related membranes (MAMs) also play an important role in the regulation of ferroptosis. By inhibiting the endoplasmic reticulum-mitochondrial sensor σ1R, the CGI1746 ligand inhibits its activity at the junction of the organelles, and finally prevents the mitochondria from uptake of Ca^2+^. By inhibiting the inflow of Ca^2+^, mitochondrial ROS production and the accumulation of polyunsaturated fatty acids (PUFA) are reduced, and the ferroptosis finally inhibited (Zhang et al., 2024) Eventually, mitochondria mediate ferroptosis through double regulatory factors. AMPK regulates ferroptosis by phosphorylating downstream targets, and the phosphorylation of the core autophagy Beclin-1, which is driven by it, promotes ferroptosis by inhibiting the activity of the XC^−^ system (Song et al., 2018). Therefore, the production of mitochondrial metabolism and reactive oxygen (ROS) are multiple factors that facilitate ferroptosis.

## 4 Ferroptosis defense systems

### 4.1 SLC7A11-GSH-GPX4 axis

The main barrier that inhibits the path of ferroptosis is the redox of the cell, which is that it can instantly remove membrane phospholipid peroxide. Among them, the SLC7A11–GSH–GPX4 axis with amino acid metabolism as the core is the main mechanism of anti-ferroptosis at present. Under the action of XCˉ, the light-chain transporter protein XCT (SLC7A11) binds to the heavy-chain protein SLC3A2 to form a heterogeneous dimer cysteine/glutamic acid exchanger (Sato et al., 1999; Koppula et al., 2021). The inflowing cysteine is reduced to methyl cysteine under the electrons provided by trioxygen reductase 1 (TXNRD1). With cysteine as the substrate, the cascading reaction of two common enzymes for the synthesis of antioxidant glutathione (GSH). One is glutamyl cysteine synthase (GCL) and the other is glutathione synthase (GSS). Under the action of the two enzymes, it is connected to glutamic acid and glycine to synthesize antioxidant glutamine cysteine (GSH). As a highly efficient electron body, glutathione (GSH) also provides an essential substrate for the activation of GPX4 enzyme, enabling the enzyme to convert harmful PL-OOHs into harmless PL-OHs, thus inhibiting the occurrence of ferroptosis (Ursini and Maiorino, 2020).

As an antioxidant enzyme that repairs lipid membranes, GPX4 constitutes the main barrier against ferroptosis. GPX4 can not only reduce lipid peroxide (L-OOH) to harmless lipiodols for detoxification, but also oxidize two molecules of glutathione (GSH) into glutathione desulphated (GSSG) (Ursini et al., 1982; Seibt et al., 2019). Previous studies have shown that mutation or inactivation of GPX4 can trigger ferroptosis so it is believed that the enzyme plays a key role in the process of ferroptosis (Yang et al., 2014; Friedmann Angeli et al., 2014). The activity of GPX4 is regulated by multiple layers, such as epigenetic regulation, transcription regulation and post-translational regulation, among which these regulatory processes involve phosphorylation, ubiquitination, succination and glycosylation (Cui et al., 2022).

### 4.2 The FSP1-CoQ10-NAD(P)H system

A 2019 study showed that FSP1 teams up with CoQ10 to create a ferroptosis defense axis that operates independently of GPX4. This FSP1–CoQ10 pathway provides the antioxidant defense required to prevent ferroptosis when GPX4 is missing (Zhu et al., 2024). The protein AIFM2, which has been renamed FSP1, exhibits a variable mitochondrial localization. This is notable because, unlike AIF, it does not possess an N-terminal mitochondrial targeting sequence. Once reisolated, FSP1 gains potent ferroptosis-suppressing activity. FSP1 operates as a flavin-linked reductase, funneling electrons from NAD(P)H to ubiquinone and thereby generating the antioxidant ubiquinol (CoQ10-H_2_). This regenerated CoQ10-H_2_ neutralizes lipid peroxidation radicals, thereby blocking both lipid peroxidation and ferroptosis.

Under certain conditions, FSP1 prevents ferroptosis not through its oxidoreductase activity, but by triggering ESCRT-III-mediated membrane repair. The ESCRT-III complex patches plasma-membrane lesions, delaying the lethal rupture that drives ferroptosis. Recent work shows that blocking the oncogenes MDM2 and MDMX boosts FSP1 expression. This elevation is orchestrated by the nuclear receptor PPARα, a master switch of peroxisome proliferation. A cell-surface safeguard built on FSP1, CoQ10 and NAD(P)H shields the membrane from injury. against ferroptosis that operates independently of GPX4. Blocking FSP1 could therefore provide a promising therapeutic strategy for AD and cancer by unleashing ferroptosis.

### 4.3 The GCH1-BH4 system

Recent studies reveal that GCH1-mediated BH4 metabolism controls lipid peroxidation during erastin-induced ferroptosis. Kraft’s team fingered the rate-limiting BH4-maker GCH1 as a potent ferroptosis brake through a CRISPR/dCas9 overexpression screen. GCH1 governs the first and bottleneck reaction of BH4 production, generating the requisite cofactor for aromatic amino-acid oxygenase. High GCH1 levels have been shown to selectively guard phospholipids that carry two PUFA tails against peroxidation. BH4 can also modulate lipid peroxidation pathways linked to coenzyme Q10 by influencing the synthesis of its precursors through interference with the phenylalanine-to-tyrosine conversion. Hence, the GCH1– BH4 axis constitutes another key defense against ferroptosis.

### 4.4 The DHODH-CoQH2 system

The DHODH-driven CoQH2-generating relay, newly mapped to the inner mitochondrial membrane, acts as a novel checkpoint that halts the ferroptosis. that operates independently of GPX4; by regeneration CoQH2, it compensates for GPX4 loss and neutralizes lipid peroxidation inside mitochondria. Embedded at the inner mitochondrial membrane, the pyrimidine-biosynthetic reductase DHODH transfers electrons to CoQ10, yielding its antioxidant hydroquinone form CoQH2, mirroring FSP1’s antioxidant role on the outer membrane. Once GPX4 is lost, flux through DHODH surges, boosting CoQH_2_ production inside mitochondria. This renewed CoQH_2_ neutralizes lipid radicals, halting mitochondrial lipid peroxidation and preventing ferroptosis. Simultaneous deletion of the mitochondrial guardians GPX4 and DHODH ignites rampant membrane-lipid oxidation, driving robust ferroptosis. Cultures skimping on GCH1 or DHODH swiftly succumb to the ferroptosis, while those amassing either enzyme gain robust armor against it.

## 5 New bioinformatics analysis based on genes associated with ferroptosis

Thanks to the rise and application of bioinformatics, a growing number of previously unknown genes associated with ferroptosis are constantly being identified. Studies have shown that transcription factors P53 and Nrf2 are also crucial regulators of ferroptosis (Jiang et al., 2015; Sun et al., 2016). Herein, we summarize the key ferroptosis-related genes and elaborate on how they regulate this cell death pathway.

P53 impairs the cystine/glutamate antiporter by silencing SLC7A11, its key component, resulting in cellular cysteine depletion and triggering ferroptosis. Nulton - 3, an MDM2 antagonist, stabilizes p53 via a p21 - dependent pathway. This stabilization enables cells to resist metabolic damage such as cysteine starvation by regulating the abundance of glutathione (GSH) (Jiang et al., 2015). Conversely, P53 inhibits dipeptidyl peptidase 4 (DPP4), thereby blocking the iron-catalyzed lipid peroxidation cell death program that erastin attempts to activate. When P53 is absent, DPP4 can freely bind to NOX1 and assemble a NOX1–DPP4 complex that drives ferroptosis. This pair mediates the formation of lipid free radicals on the outer membrane, committing cells to ferroptosis (Jiang et al., 2015).

BECN1 is a key autophagic factor that initiates autophagosome formation in the early stage of autophagy. It has been identified as a regulator of ferroptosis. In cells undergoing erastin or sulfasalazine-induced ferroptosis, AMPK phosphorylates BECN1, which then docks to XCT, the light-chain transporter within system XC^−^, forming the BECN1–XCT module. Inhibition of system XC^−^ activity blocks cystine uptake, thereby triggering ferroptosis (Guo et al., 2020). The selection of phosphorylation sites determines whether BECN1 enters the BECN1–XCT complex and triggers ferroptosis. The BECN1–XCT module also induces an iron-dependent cell death cascade in SH-SY5Y neuroblastoma cells (Chen et al., 2019).

NRF2 preserves redox homeostasis and inhibits ferroptosis: via the sequestosome-1–Keap1–NFE2L2 signaling pathway, it induces genes that limit iron and reactive oxygen species (ROS), such as NQO1, HO1, and FTH1. Further studies have identified SLC7A11 as a gene directly transcriptionally activated by NRF2. Therefore, genes like SLC7A11 may also contribute to NRF2-mediated protection against ferroptosis and warrant further investigation (Sun et al., 2016).

FANCD2, a nuclear DNA-damage-repair protein, has newly been fingered as a ferroptosis gatekeeper. Song’s team revealed that loss of FANCD2 triggers ferroptosis in bone-marrow stromal cells (Brinkmann et al., 2004). One consideration, they observed that FANCD2 deletion markedly reduces FTH1 and STEAP3, a metal reductase that converts Fe^2+^ to Fe^3+^, thereby disrupting intracellular free-iron handling akin to FTH1. Another consideration, in marrow stromal populations lacking FANCD2 expression, GPX4 mRNA dipped modestly, yet its protein level was sharply cut. These observations imply that FANCD2 modulates protein levels via both transcriptional and non-transcriptional routes. The two distinct mechanisms converge to raise intracellular Fe^2+^ and exhaust glutathione. In a word, FANCD2 regulates ferroptosis by modulating intracellular iron metabolism.

Selenium is an essential trace element in the human body. The process of inhibiting ferroptosis is to promote the transcription of GPX4 by cooperating with TFAP2c and SP1, thus maintaining the active protective neurons of GPX4 (Alim et al., 2019).

NFE2L2 mainly protects genes against oxidative damage caused by ferroptosis through transcriptional upregulation, and its signal axis becomes a key defense mechanism against ferroptosis. The genes regulated by NFE2L2 are mainly responsible for regulating iron homeostasis, glutathione metabolism and the antioxidant defense of cells against reactive oxygen (ROS).

## 6 Distinguishing ferroptosis from alternative cell-death modalities

Physiological morphogenesis and the genesis of novel disorders alike hinge on the regulated elimination of cells. The demise of cells is chiefly parsed into two operational categories: accidental cell death (ACD) and regulated cell death (RCD) (Han et al., 2020). Accidental cell death (ACD) is defined by rapid, catastrophic cell demise that occurs immediately after severe chemical, physical, or mechanical damage. In comparison, RCD advances along preset signaling networks and can be expedited or stalled through deliberate gene editing or drug treatment (Galluzzi et al., 2018). In 1970, histopathologists initially categorized lethal cellular outcomes into three archetypes, apoptosis, autophagy, and necrosis (Schweichel and Merker, 1973). Guided by structural fingerprints and signaling circuitry, regulated cell death is split into canonical caspase-powered self-destruction and a spectrum of lethal programs that operate outside the apoptotic blueprint (Tang et al., 2019).

Programmed self-elimination exhibits clumped nuclear DNA, fragmented nuclei, intact plasma membranes, shrunken cytoplasm, and disassembly into membrane-sealed vesicles known as apoptotic bodies. This form of cell death is executed through activation of the caspase cascade (Redza-Dutordoir and Averill-Bates, 2016). It was not until 2012 that Stockwell’s team first described and coined the term ferroptosis for this a lethal program fueled by catalytic iron and driven by lipid peroxidation, which is marked via a surge in membrane-bound reactive oxygen radical (Dixon et al., 2012). In 2018, the NCCD defined ferroptosis as a GPX4-dependent, iron-catalyzed lethal program triggered by intracellular redox imbalance and counteracted by lipid-soluble radical scavengers and iron-chelating agent (Galluzzi et al., 2018).

Unlike apoptosis, necroptosis, or any other cell-death route, ferroptosis is exclusively fueled by the iron-catalyzed accumulation of phospholipid hydroperoxides. Morphologically, ferroptosis exhibits mitochondria that appear shrunken and hyper-dense, featuring thickened membranes, depleted or missing cristae, and a breached outer envelope (Wang et al., 2020). At the metabolic-chemistry level, oxidative stress drives the Fenton reaction, in which ferric ions spark the peroxidative remodeling of membrane PLs into their hydroperoxide counterparts (PL-OOHs) (Li et al., 2020).

## 7 Ferroptosis in brain microenvironment

**FIGURE 3:**
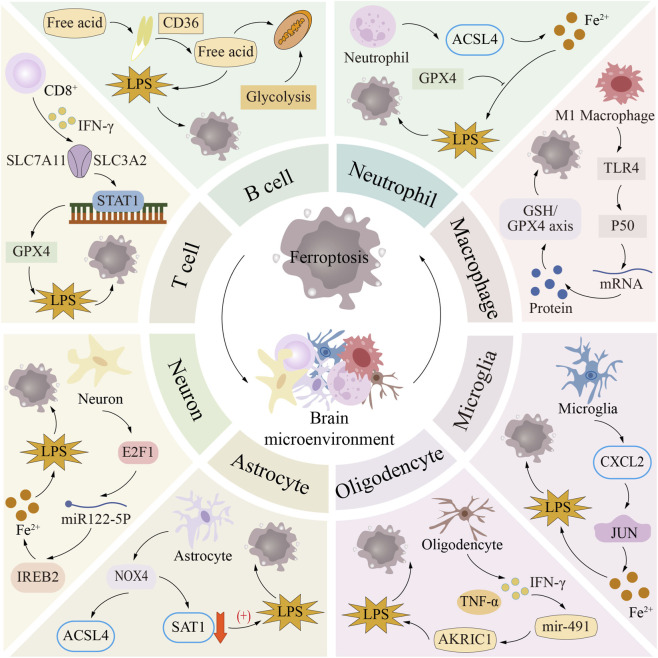
Ferroptosis in brain microenvironment. The core components of the brain microenvironment include nerve cells and neuroimmune cells. Among them, neuroimmune cells (such as T cells, B cells, neutrophils and macrophages) and nerve cells (such as glial cells and neurons) play key roles in neuroimmune regulation and neurodegenerative diseases, respectively. The profound effects and complex regulatory mechanisms of ferroptosis in these cells are closely related to the occurrence and development of a variety of diseases.

The brain microenvironment consists primarily of neural and neuroimmune cells. Immune cells contribute to disease development, inflammation, tissue injury, and repair. In neuroimmune disorders like MS and NMO, T and B lymphocytes are the key immune culprits that ignite inflammation and inflict damage within the central nervous system (Jarius et al., 2020; Jakimovski et al., 2024). Similarly, robust immune cell responses are now recognized to promote the pathological progression of neurodegenerative syndromes such as AD (Sabatino et al., 2019; Gao et al., 2023). In both neuroimmune and neurodegenerative disorders, cells of both immune and neural lineages—adaptive lymphocytes (T and B), innate phagocytes (neutrophils, macrophages) plus resident nervous-system populations (astrocytes, microglia, neurons)—constitute indispensable collaborators. Numerous studies have shown that the process of ferroptosis has a profound impact on these cells and is regulated by them so complexly that it affects a wide range of disease areas (Figure 3).

### 7.1 T lymphocyte

T cells develop from precursors of bone marrow pluripotent stem cells, acquire immunocompetence in the thymus, and differentiate into distinct subsets such as helper, cytotoxic, and activated T cell effectors. These T cells are key participants in cell-mediated immunity. However, ferroptosis disrupts the delicate balance of T cell homeostasis (Spetz et al., 2019). First, when T cells undergo ferroptosis, their ability to mount effective immune responses against infections is impaired. A 2015 study demonstrated that in the absence of GPX4 regulation, CD4^+^ and CD8^+^ T cells fail to respond to acute lymphocytic choriomeningitis virus (LCMV) or acute helminth/protozoan invasion (Matsushita et al., 2015).

First, Lipid peroxidation-driven ferroptosis in T cells impairs their ability to combat infections. Second, Drijfhout et al. demonstrated that CD8^+^ T cells can be rescued from ferroptosis when FSP1 or GPX4 is overexpressed, or when ACSL4 is deleted (Drijvers et al., 2021). Furthermore, the crosstalk between T cell-mediated immunity and ferroptosis is crucial for the efficacy of cancer immunotherapy (Matsushita et al., 2015). CD8^+^ T cells secrete interferon-γ (IFN-γ), which downregulates the expression of SLC3A2 and SLC7A11 in the XC^−^ system. This inhibition disrupts lipid oxidation control within tumor cells, thereby inducing ferroptosis (Lang et al., 2019). Furthermore, tumor-infiltrating CD8^+^ T cells exhibit extensive lipid peroxidation, which is apparently triggered by fatty acid action, thereby increasing CD36 levels and driving the cells into ferroptosis (Ma et al., 2021). On the contrary, regulatory T cells (Tregs) drive ferroptosis by promoting the secretion of antioxidant thioxygen-1 (Trx-1) (Mougiakakos et al., 2011). Although the number of regulatory T cells (Tregs) lacking GPX4 is reduced, it secretes a higher level of interleukin-1β (IL-1β) in the tumor microenvironment, thus enhancing the Th17 response to inhibit tumor growth.

### 7.2 B lymphocyte

B lymphocytes, that is, what we often call B cells, originate from pluripotent hematopoietic proctor cells in the bone marrow cavity. These processor cells will differentiate into different subgroups, such as B-1 lymphocytes, conventional B-2 groups, follicular B lymphocytes, marginal B cells, and immunosuppressive regulatory B cell subgroups. When encountering the corresponding antigen, B lymphocytes will mature and produce corresponding antibodies to protect the body from damage. GPX4 is equally critical to B cell biology, mirroring its indispensable role in T cells. Distinct B cell subtypes display varying vulnerability to ferroptosis. When GPX4 is poorly expressed, B1 as well as marginal-zone B cells accumulate lipid peroxides and readily undergo ferroptosis. Yet, follicular B cells remain immunologically competent even when GPX4 is absent. Pharmacological blockers of GPX4 have been demonstrated to trigger ferroptosis in B cells within the context of central-nervous-system mitochondrial diseases (Kahn-Kirby et al., 2019).

Protein–protein interaction networks also point to possible connections between ferroptosis and both IgA production and B cell receptor signaling (Criscitiello et al., 2020). Research published in 2018 proposed that autophagy coupled to ferroptosis could fine-tune B cell function (Clarke et al., 2018). Ferroptosis is also being explored as a druggable axis for disorders rooted in B-lineage lymphocytes. Within a murine experimental system mimicking DLBCL, blocking ferroptosis triggered by the System XC^−^–GPX4 pathway markedly slowed tumor growth and progression. Consequently, this tactic unveils an innovative treatment route for the aggressive B cell malignancy known as DLBCL (Kinowaki et al., 2018).

### 7.3 Neutrophil

As frontline defenders against invading pathogens, neutrophils provide essential protection by phagocytosing microbes, releasing granule contents, and forming neutrophil extracellular traps (NETs) (Papayannopoulos and Zychlinsky, 2009). Ferroptosis governs both the recruitment and the immunomodulatory activity of neutrophils during inflammatory responses (Chen et al., 2022a). Recent work shows that ferroptosis summons polymorphonuclear granulocytes to zones of non-infectious myocardial insult following cardiac grafting., thereby triggering an inflammatory response. However, administration of the ferroptosis inhibitor ferrostatin-1 (Fer-1) markedly suppresses neutrophil recruitment and reduces cardiac muscle cell death (Li et al., 2019).

Furthermore, ferroptosis appears to be associated with the release of neutrophil extracellular traps (NETs) and the death of neutrophils. Upon stimulation, neutrophils release complex structures composed of DNA strand networks studded with bactericidal peptides, which are termed neutrophil extracellular traps (NETs) (Brinkmann et al., 2004). Sul pyridine is an ferroptosis inducer. Its mechanism of action is mainly to promote the formation of NETs through ether phospholipid peroxide and promote the entry of neutrophils into ferroptosis (Yotsumoto et al., 2017). In addition, the ferroptosis of neutrophils is closely related to anti-cancer therapy. Researchers found neutrophils undergoing ferroptosis in the tumor microenvironment of colorectal cancer liver metastasis (CRLM) through single cell RNA sequencing (scRNA-seq) technology (Zhang et al., 2020). The most important thing is that research shows that neutrophil-mediated ferroptosis accelerates necrosis in glioblastoma masses (Yee et al., 2020). In addition, a study in 2022 showed that ferroptosis can selectively inactivate abnormally activated neutrophils, thus eliminating their inhibitory effect against tumor immunity, thereby inhibiting the tumor progression of immune-healthy animals (Kim et al., 2022).

### 7.4 Macrophage

Arising from monocytes, macrophages eliminate pathogens through their robust phagocytic activity and are central to maintaining immune balance. Two distinct macrophage subtypes are recognized—M1, known for promoting inflammation, and M2, recognized for resolving inflammation. Within the intricate control of GPX4, M1 and M2 macrophages show contrasting vulnerability to ferroptosis triggered by GPX4 inhibitors (Kapralov et al., 2020). Upregulated inducible nitric oxide synthase (INOS) strengthens M1 macrophages against ferroptosis (Kapralov et al., 2020). Deleting SLC7A11 sensitizes bone-marrow derived macrophages to ferroptosis (Wang et al., 2017). Excessive erythrophagocytosis by macrophages promotes ferroptosis in murine systems modelling RBC transfusion and removal (Youssef et al., 2018). In opposition, Geng and colleagues reported that the inflammatory-macrophage metabolite itaconate curbs sepsis-linked lung damage by suppressing macrophage ferroptosis via NRF2 signaling (He et al., 2022) A 2022 study first showed that igniting iron-catalyzed lipid-peroxidative death inside immune scavengers sharpens their germ-killing grip on invaders; follow-up rodent trials with ferroptosis-boosting drugs then proved that this oxidative storm speeds microbial purge (Ma et al., 2022).

Through inducing ferroptosis in cancer cells, radiotherapy redirects M2 macrophages toward an M1 phenotype during antitumor treatment (Wan et al., 2020). Moreover, macrophages detect and phagocytize siderophiles with peroxidized PE lipids studding their outer leaflet via Toll-like receptor 2 (TLR2), which accelerates their disposal (Luo et al., 2021). Within cancer therapy, TLR2 drives macrophages to phagocytose iron-tropic tumor cells, which in turn establishes a sturdy springboard for braking malignancy’s March along iron-dependent avenues.

### 7.5 Microglia

Brain-resident macrophages engage in debris engulfment and cytokine release, steering innate defense within CNS insults, ultimately tipping the balance toward either neuronal rescue or damage (Prinz et al., 2019). Once damage cues switch on, microglia cells split into the pro-inflammatory M1 camp and the tissue-mending M2 camp, thereby executing both offensive and defensive immune roles. Notably, recent studies have shown that microglia contribute to the precise regulation of metal homeostasis and oxidative stress in the central nervous system (CNS). Iron accumulation in the CNS is induced by activated microglia (Guo et al., 2021). It can stimulate the activation of microglia, upregulate the expression of ferritin, and release immune-activating signaling molecules such as interleukin-1β (IL-1β), interleukin-6 (IL-6), and tumor necrosis factor-α (TNF-α) (Healy et al., 2016; Wa et al., 2013).

Nevertheless, ferroptosis-inhibiting compounds represented by Ferrostatin-1 (Fer-1) and Liproxstatin-1 can promote M2 polarization of microglia, alleviate inflammation, and suppress the release of immune-activating messenger molecules (Huang et al., 2022). Another study demonstrated that excessive heme oxygenase-1 (HO-1) production in microglia triggers harmful iron accumulation, which can be effectively controlled and corrected by iron chelators such as deferoxamine (DFX) (Fernández-Mendívil et al., 2021). Besides, the production of excessive reactive oxygen (ROS), the depletion of glutathione (GSH) and the production of lipid peroxidation together contribute to the ferroptosis of small glial cells (Wu et al., 2020). It is worth noting that the accumulation of reactive oxygen (ROS) in the overactivated central nervous system (CNS) may lead to the gradual destruction of dopaminergic neurons, which is mainly catalyzed by NADPH oxidase (Sharma and Nehru, 2016). In addition, in the process of inflammation of small glial cells, induced nitric oxide synthase (iNOS) can inhibit ferroptosis by inhibiting the activity of 15-lipooxygenase (15-LOX) (Kapralov et al., 2020).

### 7.6 Astrocyte

Astrocytes are widely distributed in the central nervous system (CNS), mainly responsible for communicating with neighboring nerve units, maintaining the homeosis of the nervous system, and participating in the formation of the blood-brain barrier (BBB) (Lee et al., 2022). Astrocytes also regulate brain metal homeostasis by transporting transferrin-bound iron and heme-bound iron (Xu et al., 2017). Astrocytes acquire iron via the divalent metal transporter 1 (DMT1) pathway, while ceruloplasmin located on the astrocyte membrane effectively inhibits lipid peroxidation and ferroptosis through protein-protein interactions or regulating the oxidation of Fe^2+^ to Fe^3+^ (Jeong and David, 2003).

Furthermore, elevated heme oxygenase-1 (HO-1) levels in astrocytes promote iron accumulation in the striatum—a burden that can be reversed by the metal chelating drug deferiprone (DFP) (Song et al., 2017). On the other hand, ferroptosis induced by the accumulation of reactive oxygen (ROS) and the depletion of glutathione (GSH) and glutathione peroxidase 4 (GPX4) in astrocytes can be regulated by NRF2/HO-1 cascading reaction. In addition, this process can also be strongly inhibited by Ferrostatin-1(Fer-1). Ferrostatin-1 (Fer-1) (Li et al., 2021a). A variety of antioxidant molecules secreted by reactive astrocytes, such as glutathione (GSH), metallothionein’s (MTs), and nuclear factor erythroid 2-related factor 2 (Nrf2), act as key defenders regulating oxidative stress and metal homeostasis, thereby conferring neuroprotective effects (Wang et al., 2022). The role of astrocytes in coping with oxidative stress during ferroptosis provides a novel approach for the treatment of ferroptosis-related diseases.

### 7.7 Oligodendrocyte

Within the brain and spinal cord, oligodendrocytes spin insulating lipid layers around axons, speed up electrical messaging, and stand guard to keep neurons working smoothly. Oligodendrocyte precursor cells (OPCs) are characterized by their capacity to multiply and relocate, while also serving pivotal parts in advancing CNS pathologies. Because iron is indispensable for wrapping myelin and powering metabolic enzymes, oligodendrocytes outrank every other CNS cell population in intracellular iron content (Friedrich et al., 2021; Jhelum et al., 2020). Ferritin-mediated ferroptosis has been demonstrated to cause oligodendrocyte loss and demyelination in murine versions of cuprizone-triggered MS-like demyelination (Jhelum et al., 2020).

Iron is indispensable for myelin genesis, yet through Fenton chemistry it generates hydroxyl radicals that trigger lipid peroxidation and further iron buildup. Fan et al. showed that blocking ferroptosis with agents such as Fer-1 and Liproxstatin-1 fosters OPC maturation and myelin production by easing mitochondrial oxidative pressure and lipid peroxide buildup (Fan et al., 2021). Furthermore, certain studies have shown a close relationship between oligodendrocytes and oxidative stress-induced injury. Ferroptosis can be triggered by inhibiting the cystine–glutamate antiporter System XC. or oxidative stress injury, leading to myelination or demyelinating damage (Li et al., 2022).

### 7.8 Neuron

Neurons serve as the essential building blocks and functional elements of the central nervous system. Glia sustain brain metabolic equilibrium via interactions among astrocytes, oligodendrocytes, and microglia. GPX4-governed restraint of ferroptosis indispensable for safeguarding movement-commanding neurons within the central nervous system (Schriever et al., 2017). Essential ferroptosis mechanisms, such as membrane lipid oxidation coupled with mitochondrial failure, which are linked to neuronal degeneration in the cerebral cortex, are efficiently decelerated by GPX4 (Chen et al., 2015). Insufficient GPX4 in dopaminergic neurons leads to anxiety-like behavior (Schriever et al., 2017). These glial cell types are associated with iron-dependent, lipid-peroxidative cell death that stems from perturbed metal homeostasis.

Additionally, excess metal can be shuttled into nerve cells, provoking iron-mediated neurotoxicity. Iron has been shown to bind α-synuclein and travel from cell to cell via membrane nanotubes, vesicle internalization, and exosome-mediated delivery. In a similar manner, nerve cells likewise undergo redox strain during senescence.

In 2021, the research team led by Martin Kampmann revealed neuron-specific ferroptosis pathways through the study of ROS regulation in human neurons. Studies reveal that the lysosomal factor PSAP governs reactive oxygen species homeostasis and highlight its role in triggering neuronal iron-dependent death by modulating lysosomal lipid handling (Tian et al., 2021). Furthermore, a 2022 study revealed that targeting the thrombin-ACSL4 axis may help reduce ferroptosis in neurons damaged by ischemic stroke (Tuo et al., 2022).

## 8 The mechanisms of ferroptosis in AD

**FIGURE 4:**
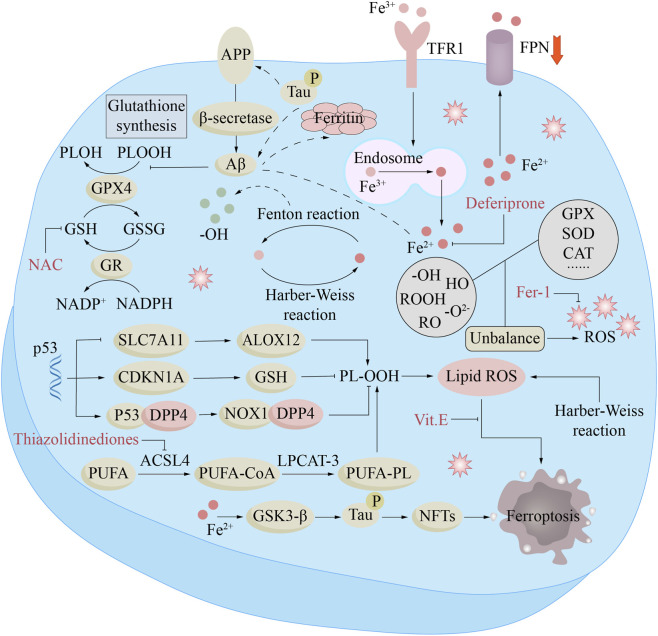
Ferroptosis mechanisms and treatment in Alzheimer’s disease (AD). In AD, Fe^2+^ enhances β-secretase activity by reducing furin protein expression, thereby promoting Aβ production through the amyloidogenic pathway. The downregulation of ferroportin (Fpn) is directly induced by Aβ, while increased intracellular liberation of Fe^2+^ activates the ferroptosis pathway. Furthermore, Aβ decreases glutathione peroxidase 4 (GPX4) levels and elevates ferritin expression. Excessive iron in neurons can lead to Tau hyperphosphorylation and the formation of neurofibrillary tangles (NFTs) via the GSK-3β kinase pathway. Compounds such as N-acetylcysteine (NAC), thiazolidinediones, ferristatin-1 (Fer-1), α-tocopherol (Vitamin E), and deferiprone target different components of this pathway to inhibit ferroptosis, which may underlie their potential clinical benefits in AD.

AD stands as the most widespread neurodegenerative illness, marked by brain shrinkage, aggregation of Aβ fragments into extracellular plaques and clustering of abnormally phosphorylated tau into intraneuronal tangles drive the stepwise erosion of mental capacity (Hardy and Higgins, 1992). Key manifestations in individuals with this condition encompass consistent memory troubles, indistinct speech, sluggish routine tasks, mood swings, and challenges in comprehending issues and commands (Lyketsos et al., 2011). Despite the toxic accumulation of Aβ being considered the main driver of Alzheimer’s pathology, clinical strategies aimed at lowering Aβ levels have so far failed to meaningfully slow disease advancement (Morris et al., 2014). In the past few years, the ferroptosis–AD link has been extensively probed. Research has shown that the control of ferroptosis following AD is associated with various pathways. These pathways involve iron metabolism disruption pathways and L-ROS accumulation in lipid metabolism pathways. They further encompass diminished GPX4 and GSH concentrations within redox-regulating routes, alongside multiple additional non-canonical signaling cascade (Chen et al., 2021b). In this review, we clarify the mechanisms of ferroptosis in AD (Figure 4).

### 8.1 Iron metabolism dysregulation

Iron is unevenly distributed in the cerebral cortex: the metal burden in motor-related nuclei—including the basal ganglia, amygdala, substantia nigra, hippocampus, and red nucleus—is significantly higher than in other neural regions (Bartzokis et al., 2007). It is generally accepted that aging leads to brain iron accumulation, but whether this metal accumulation is a cause of neurodegenerative diseases or merely reflects neuronal death remains unclear (Sato et al., 2022). The elevated iron content in the brains of patients with Alzheimer’s disease was first documented in 1953 (Goodman, 1953). Subsequently, studies have traced a direct pathway from cerebral iron overload to neuronal death and cognitive decline in patients with AD (Spotorno et al., 2020; Ayton et al., 2021). Ayton and his colleagues demonstrated that elevated metal levels in the inferior temporal lobe promote glial cell activation, redox imbalance, and iron-dependent neuronal death, thereby accelerating memory decline in patients with Alzheimer’s disease (Ayton et al., 2020).

Further research by Qin’s team has shown that increased metal burden in the hippocampus and parietal lobe regions correlates with the severity of cognitive deterioration in Alzheimer’s disease subjects. These findings establish the potential of iron as a candidate biomarker for tracking the progression of AD (Qin et al., 2011). Redox-active iron has been detected in the cerebella of individuals with preclinical AD pathology, and its levels increase as patients’ cognitive function declines (Smith et al., 2010). Hansra and colleagues have mapped the increased iron deposition in the hippocampus, parietal lobe and motor areas of Alzheimer’s disease tissues, where the deposition sites coincide with neurofibrillary tangles and amyloid plaques. These findings are highly congruent with both the rate of neuronal loss and the severity of cognitive decline in Alzheimer’s disease patients (Hansra et al., 2019).

Elevated iron levels occur even in the early stages of AD, presenting as mild cognitive impairment (MCI). In mouse models of AD, superfluous unchaperoned iron settles within senile plaques, escalates ferritin synthesis, and blunts spatial recall (Peters et al., 2018a; Peters et al., 2018b). APP, a membrane sentinel, detoxifies iron overload by charging transferrin with Fe^3+^ and securing FPN stability (Duce et al., 2010). Animals lacking APP display diminished FPN excess cortical iron deposition, and heightened neuronal oxidation (Duce et al., 2010).

Elevation of the iron accelerates APP synthesis via the IRE switch; once bound, iron amplifies the toxic punch of both Aβ and tau. FPN depletion causes memory decline by inducing ferroptosis in AD patients (Bao et al., 2021). FPN expression was diminished in both APP Swe/PS1dE9 Alzheimer-model mice and human AD brain tissue. FPN depletion in rodents drives hippocampal wasting and recall failure that replicate AD-type cognitive decay. In cell dishes and living brains alike, blocking ferroptosis markedly curbed neuron loss and memory impairment triggered by Aβ clump. Restoration of FPN ameliorated ferroptosis and memory impairment in APP Swe/PS1dE9 mice (Bao et al., 2021). The evidence indicates that disruption of cerebral iron equilibrium can ignite neurodegenerative cascades culminating in Alzheimer’s pathology.

### 8.2 Lipid metabolism pathway in AD

L-ROS buildup and lipid metabolism disruptions are implicated in Alzheimer’s pathogenesis. Lipid peroxidation relies heavily on polyunsaturated fatty acids (PUFAs) as fundamental reactants, and there is evidence of heightened consumption and metabolism of PUFAs, specifically, arachidonic acid (AA) and adrenic acid (AdA) are prominent in the neural tissues of those suffering from Alzheimer’s disease (Czapski et al., 2016). The accumulation of AA in the brains of Alzheimer’s disease patients and in animal models implies its role in the development of Alzheimer’s disease. In cellular and murine models of Alzheimer’s disease, AA and its derivatives have been identified as associated with the generation of Aβ40 and Aβ42 peptides and the development of Alzheimer’s disease by Amtul and colleagues (Amtul et al., 2012). Thomas et al. reported that dietary intake of arachidonic acid (AA) in mature male BALB/c rodents triggers memory decline and β-amyloid-mediated neuronal injury, potentially advancing toward Alzheimer’s pathology. Dietary uptake of arachidonic acid (AA) remodeled the hippocampal and cortical lipid signature and phosphatide mapping. Investigators additionally uncovered that subjects maintained on an AA-loaded regimen showed suppressed ACSL4 signal within the hippocampal sector, coupled with intensified astroglia scarring and elevated cytoplasmic PLA2 turnover across the cortical sheet (Thomas et al., 2017).

ACSL4 is associated with neurological diseases and is indispensable for the activation of long-chain polyunsaturated fatty acids (PUFAs) as well as iron-driven cell death cascades (Shin et al., 2018). ACSL4 is widely expressed in neural tissues, particularly in the CA1 region of the hippocampus, and its levels continue to increase with the progression of neurodegenerative diseases, leading to cognitive decline (Gubern et al., 2013). Lipid peroxidation decomposition produces a series of carbonyl metabolites—such as 4-hydroxynonenal (4-HNE), malondialdehyde (MDA), and acrolein (Dang et al., 2010). Previous studies have documented elevated lipid peroxidation (LPO), decreased polyunsaturated fatty acids (PUFAs), and increased 4-hydroxynonenal (4-HNE) in the cerebrospinal fluid (CSF) of patients with AD, along with excessive oxidative stress markers in brain tissues (Prasad et al., 1998; Pettegrew et al., 2001). Elevated levels of 4-hydroxynonenal (4-HNE) and acrolein are also observed in the early stages of MC and AD-type dementia, indicating that lipid oxidation is an initiating event in the pathogenesis of AD (Bradley et al., 2010).

Early studies have shown that specific catalysts driving fatty acid oxidation—including lipoxygenases (LOXs), cyclooxygenases (COX), and NADPH oxidases (NOXs)—are upregulated in AD pathology and play a key role in its progression. A recent investigation revealed that NOX4, a chief factory of redox radicals, was strikingly upregulated within scarred astroglia populating the cortical mantle of human Alzheimer cases and APP/PS1 dual-transgene mouse replicas. Excessive NOX4 expression triggers ferroptosis through lipid peroxidation activation in human astrocytes, enhancing cytotoxic effects (Park et al., 2021). In summary, disordered lipid oxidation homeostasis and perturbed fat-handling cascades jointly propel facets of Alzheimer’s pathogenesis.

### 8.3 GSH/GPX4 in AD

Research has revealed that diminished glutathione (GSH) concentrations within the hippocampal and prefrontal regions align with pronounced memory impairment, pointing to GSH as a potential AD signature (Ayton et al., 2020). Oral administration of supplements to restore brain glutathione and l-cysteine levels proves utterly fruitless given that GSH and l-cysteine undergo rapid degradation and display only partial transit across the cerebral vasculature barrier. Nevertheless, the l-cysteine prodrug N-acetylcysteine (NAC) efficiently crosses the cerebral endothelium and permeates into brain parenchyma. NAC mitigates lipid peroxidation in the brain through modulating cortical glutathione content in a transgenic rat mimic of Alzheimer’s pathology. NAC can boost cell and mitochondrial membrane permeability, thus raising GSH levels, suppressing ferroptosis, and providing neuroprotection (Terluk et al., 2019; Tardiolo et al., 2018).

Loss of GPX4 function in anterior-brain neurons drives cell loss within the hippocampal region. In the aquatic navigation test, GPX4-null rodents with forebrain-restricted knockout exhibited pronounced memory deficits and substantial hippocampal nerve injury. Thioacetate halts the ferroptosis cascade by repressing transferrin receptor while boosting GPX4 translation, thereby protecting neurons associated with learning and memory (Zhang et al., 2018). New research reveals that the loss of iron-mobilizing carriers, by fueling the ferroptosis cascade, triggers cognitive decline in Alzheimer’s disorder (Bao et al., 2021). Amplified GPX4 expression protected neocortical neurons from β-amyloid–elicited death by curbing membrane lipid oxidation (Ran et al., 2006). GPX4 deletion led to neuronal death in the hippocampal area of neonatal and adult mice, along with increased astrocyte activation, indicating that GPX4 serves as a defensive sentinel during nervous-system degeneration processes (Yoo et al., 2012).

Targeted deletion of GPX4 exclusively within anterior-brain neurons triggers memory loss and hippocampal nerve cell decay in rodents (Hambright et al., 2017). In 5 × FAD mice with GPX4 overexpression (5 × FAD/GPX4), GPX4 overexpression curbed nerve cell demise and lipid-ROS surge across the prefrontal mantle, slashed β-amyloid plaque load, and sharpened cognitive performance. The 5 × FAD/GPX4 mice displayed muted ferroptosis signatures and lower 4HNE levels, supporting the idea that ferroptosis is a crucial factor in Alzheimer’s disease development (Chen et al., 2022b).

### 8.4 FSP1-CoQH2 system in AD

Novel findings show that elevated FSP1 abundance helps counteract Alzheimer’s neurodegeneration induced by chronic sleep deprivation through a ketogenic diet (Yang et al., 2022). The ketogenic diet halted brain deterioration, amyloid buildup, and tau hyperphosphorylation caused by chronic sleep deprivation (SD). The ketogenic diet also suppressed iron metabolism disruption by reducing TFR1 and DMT1 expression while increasing FTH1 and FPN1 expression. The ketogenic diet amplified the cystine–glutathione peroxidase 4 circuit, elevated ferroptosis suppressor protein 1 abundance, and curtailed malondialdehyde generation. Simultaneously, Carb-restricted ketosis switched on the Sirt1-Nrf2 relay in sleep-loss mouse hippocampal tissue. Collectively, the data indicate that the ketogenic diet (KD) may curb the ferroptosis cascade through the Sirt1–Nrf2 relay, consequently boosting GPX4 and FSP1 abundance., which in turn prevents chronic Alzheimer’s disease induced by sleep deprivation (SD) (Yang et al., 2022).

### 8.5 Ferroptosis interacts with tau protein and NFTs

The axonal protein Tau acts as a tubulin-track adaptor indispensable for microtubule docking, neuronal freight movement, and signaling network governance (Avila et al., 2004). The Tau protein is manufactured from the MAPT gene situated on the 17th chromosome (Clark et al., 1998). Variations in this DNA segment spark hereditary tauopathies—frontotemporal dementia and chromosome-17-tied parkinsonism (FTDP-17)—yet the triggers for non-familial types like Alzheimer’s remain unidentified (Wszolek et al., 2006). Post-mortem analyses reveal iron deposits trapped within tau fibrils in the cerebral cortex of Alzheimer’s victims (Smith et al., 1997).

Iron has also been shown to sway tau over-phosphorylation cascades by tuning the enzymatic action of GSK3β and CDK5 (Xie et al., 2012). In homeostatic contexts, tau migrates to the surface bilayer to accelerate cytoplasmic iron expulsion. In contrast, hyperphosphorylation and aggregation of tau can disrupt iron export, resulting in neuronal iron accumulation and worsening neurofibrillary tangles (NFTs), creating a negative feedback loop (Wang and Mandelkow, 2016). Tau’s post-translational modifications, specifically phosphorylation, can cause aggregation and neurotoxic effects. In fact, abnormally phosphorylated tau can affect both presynaptic and postsynaptic compartments, for example, by disrupting signaling pathways, mitochondrial function, and axonal transport (Soria Lopez et al., 2019).

### 8.6 Ferroptosis and pathological Aβ accumulation mediates AD

Tangled clumps of Aβ accumulating in neural tissue count as a prime hallmark of Alzheimer’s pathology. Currently, most perspectives indicate that aberrant β-amyloid bursts forth after tandem snips of membrane-bound APP by β-then γ-secretases atop neurons or ancillary cerebral cells (Stanga et al., 2018). APP fragmentation into β-amyloid fragments has been implicated in driving Alzheimer’s pathogenesis (Zhang et al., 2011). It is suggested that an overproduction of Aβ sparks peptide self-aggregation into soluble oligomers and rigid fibrillar arrays, ultimately giving rise to the amyloid deposits seen in pathological specimens (Soria Lopez et al., 2019). Amyloid-beta is suspected to fuel the lipid-peroxide burst that propels neurons into the ferroptosis.

Empirical work has revealed that lipid peroxide levels, such as 4-HNE, are markedly elevated in regions rich in Aβ oligomers, indicating that Aβ accumulation may be linked to membrane lipid oxidation (Butterfield et al., 2018). β-amyloid clusters embed within lipid leaflets, disturb hydrogen-atom stripping from membrane phospholipids during radical genesis, and modulate the onset of metal-catalyzed, enzyme-independent lipid-ROS cascades (García-Viñuales et al., 2021). Emerging research has uncovered that intraneuronal amyloid toxicity from pathological Aβ induced oxytocin/iron toxicity-regulated cell death. Therefore, Aβ, a central pathogenic output of AD, could cross-talk with membrane-lipid oxidation during the ferroptosis cascade (Huang et al., 2020).

### 8.7 Apolipoprotein E (APOE) participates in AD progression

APOE—an AD-risk determinant—operates as a vital neural lipid governor, ferrying sterols across central nervous system cells. Data reveal that APOE stockpiles acetyl-CoA—the raw material for sterol production—by slamming the brakes on rate-limiting catalysts along the cholesterol-forming cascade. Acetyl-CoA additionally acts as the carbon donor for manufacturing lipid chains rich in double bonds—PUFAs (Li et al., 2021b). Increased expression of APOE may enhance intracellular PUFAs accumulation, thereby priming the milieu for fatty-acid radical damage and the ferroptosis. Astrocytic and microglial failure is thought to fuel plaque accumulation and synapse elimination in Alzheimer’s pathology.

Nevertheless, contemporary investigations have indicated that APOE stimulates the PI3K/AKT signaling cascade to suppress ferroptosis, consequently hindering iron-catalyzed lipid peroxidation (Belaidi et al., 2024). Autopsy analyses reveal that ferric buildup tightly links to neuropathology-verified dementia of Alzheimer type, particularly among carriers harboring the ε4 variant of the APOE gene. Individuals harboring the ε4 allele of APOE display elevated lipid chains prone to oxidative attack, thereby accelerating ferroptosis. Diminished APOE abundance accelerated ferritin self-digestion and metal efflux from its storage cage, amplifying lipid radical chain reactions and iron-driven cell demise (Belaidi et al., 2024).

## 9 Targeting ferroptosis to treat AD

Up to now, drug-based interventions crafted for Alzheimer’s pathology remain non-remedial, yet they succeed in easing the clinical manifestations of the disorder (Long and Holtzman, 2019). Ferroptosis, has recently been implicated in Alzheimer’s pathogenesis and stands out as an attractive intervention point; blocking this process across different disease phases could translate into therapeutic benefit. At present, standard ferroptosis blockers chiefly operate by halting membrane-lipid oxidation, lowering labile iron pools, or curbing reactive oxygen radical generation.

### 9.1 Iron chelators

Upsetting the metal balance and buildup of loosely bound Fe^2+^ ions act as decisive triggers for ferroptosis. Hence, ferric-binding molecules have surfaced as potent tools to scavenge free Fe^2+^ and directly block the ferroptosis (Dixon et al., 2012). Sequestering excess metal is a multistep maneuver: the capturing agent must traverse the cerebral vasculature filter, selectively trap surplus mineral at local hot-spots, yet leave transferrin-tethered reserves in the bloodstream untouched and avoid shuttling them onto shuttle proteins like plasma transferrin.

The U.S. drug-regulating agency has now cleared three metal-binding compounds for alleviating clinical manifestations of neurodegenerative disease: Deferoxamine, Deferasirox (Exjade) and Deferiprone. In contrast, deferoxamine and deferasirox traverse the brain–blood partition poorly and bind iron in a dose-reliant manner, deferiprone’s molecular design enables CNS entry, making it the preferred agent in most neurodegenerative-disease clinical trials (Martin-Bastida et al., 2017; Sripetchwandee et al., 2016). Deferoxamine (DFO) is a widely utilized iron-chelating agent in clinical settings. It could be a promising therapy for AD, with DFO having been tested in animal studies and clinical trials. Indeed, studies in APP/PS1 mice fed an iron-loaded diet showed that DFO administration blocked tau hyper-phosphorylation by reining in CDK5 and GSK-3β signaling (Guo et al., 2013a). In more detail, by lowering CDK5 activity, deferoxamine triggers inhibitory phosphorylation of GSK-3β, thereby cutting tau hyper-phosphorylation. Furthermore, administering the iron chelator deferoxamine to APP/PS1 transgenic animals curbed β-amyloid generation and alleviated memory impairment (Guo et al., 2013b) In a 2-year, single-masked study of 48 dementia participants, Crapper et al. observed that intramuscular deferoxamine slowed clinical deterioration by half relative to controls. Nevertheless, deferoxamine additionally triggers reductions in body weight and food intake (Crapper McLachlan et al., 1991).

The ingestible iron-scavenger Deferasirox (Exjade), unlike its injectable counterpart, neutralizes cytosolic Fe^3+^ at a 2:1 molecular stoichiometry (Poggiali et al., 2012; Piga et al., 2006). When used in conjunction with lactoferrin (Lf), its efficacy in penetrating the cerebral vasculature wall is significantly improve (Kamalinia et al., 2013). In a recent study, Deferasirox was evaluated in three distinct murine Alzheimer paradigms—Tg2576 (APP-only), JNPL3 (Tau-only), and Tg2576/JNPL3 (APP + Tau)—to assess its therapeutic impact. Outcomes showed the intervention left memory and motor skills unchanged, yet curbed tau hyper-phosphorylation. The team proposed the agent either strips away aggregation-driving iron or docks directly onto tau to block its self-assembly (Kwan et al., 2022).

Deferiprone (DFP), an orally bioavailable iron binder, readily traverses the brain–blood partition (Hider and Hoffbrand, 2018). Within a randomized, controlled study, DFP boosted neural performance metrics and eased iron-linked brain symptoms (Abbruzzese et al., 2011; Klopstock et al., 2019). DFP is safe with minimal systemic toxicity and could serve as a promising approach for treating iron homeostasis disorders and iron poisoning in AD. Experiments on embryonic neuron cultures exposed to ferric ions, human Aβ1–40, and Aβ1–42 likewise revealed deferiprone’s nerve-sparing action. Treatment with different concentrations of deferiprone can effectively eliminate neuronal cell death (Molina-Holgado et al., 2008). It also corroborates the hypothesis that iron is a key factor in Aβ-induced neurotoxicity (Kim et al., 2018).

### 9.2 Lipid peroxidation inhibitor

ACSL4 together with lipoxygenase isoforms fuel the fatty-acid oxidation cascade that powers ferroptosis, marking them as prime docking sites for anti-peroxidation agents. Thiazolidinediones have been proven to target and inhibit ACSL4 selectively (Kim et al., 2001). TZDs sit within the PPAR-γ activator class, drugs broadly prescribed for glycemic control in type 2 diabetes. Nevertheless, in 2017, German researchers published an investigation revealed that the thiazolidinedione trio—troglitazone, rosiglitazone and pioglitazone—halt the ferroptosis demise pathway. by suppressing ACSL4 activity in both cell and animal experiment (Doll et al., 2017). Among these compounds, troglitazone achieved the most notable suppression of ferroptosis, without engaging the PPAR-γ signaling axis (Doll et al., 2017).

Beyond that, lipoxygenase isoforms are pivotal for triggering oxidative damage within polyunsaturated-fatty-acid phospholipids. Originally developed to blunt 5-lipoxygenase for asthma relief, Zileuton now proves it can selectively silence that enzyme, halt lipid-ROS propagation and shield cells from ferroptosis (Jung et al., 2023; Liu et al., 2015). PD146176, a 15-LOX–selective antagonist, additionally halts the ferroptosis cascade (Ou et al., 2016; Zilka et al., 2017). A 2021 investigation showed that α-tocopherol halts ferroptosis by shutting down arachidonate-driven LOX activity and trimming lipid-ROS output (Hu et al., 2021).

### 9.3 Antioxidants and ROS scavengers

In view of the contribution of reactive oxygen species to lipid peroxidation and ferroptosis, it makes sense to develop antioxidants and ROS scavengers to inhibit ferroptosis. In 2012, Dixon and team initially pinpointed Fer-1 emerged as the most potent blocker of Erastin-evoked ferroptosis in HT-1080 fibrosarcoma cells (Dixon et al., 2012). Ferrostatin-1 (Fer-1), a ROS-trapping agent, serves as a classic ferroptosis blocker and surpasses phenolic radical quenchers in efficacy (Miotto et al., 2020). It has been shown to blunt lipid peroxidation and block cell death across a spectrum of disease paradigms, including neurodegeneration, periventricular leukomalacia, cerebral stroke, and cardiovascular disorders.

Fer-1 quelled angiotensin-II-provoked inflammatory cascades and stellate-cell ferroptosis by dampening ROS surge and repressing Nrf2 together with GPX expression (Li et al., 2021a). Fer-1 rescues nerve cells and reverses memory loss in Alzheimer’s models, whether in live animals or cultured neurons (Bao et al., 2021). Even though robust cellular and animal data highlight Fer-1’s potency in easing oxidative injury and blocking ferroptosis, human trials have yet to commence. Its precise effectiveness and potential side effects in treating AD require further investigation. Lip-1, a mighty oxidative-stress blocker originating from spiroquinoxalinamine, potently suppresses the iron-dependent cell-death pathway known as ferroptosis (Friedmann Angeli et al., 2014). Lip-1 further sparks the NRF2 cascade, scavenges reactive oxygen species, and dampens TGF-β output, thus mitigating postradiation lung scarring and shielding the heart from injury (Li et al., 2019; Feng et al., 2019).

Using Fer-1 and Lip-1 on mouse hippocampal primary neurons stimulated by Aβ aggregation notably diminished neuronal death (Piga et al., 2006). Recent studies have shown that alpha-tocopherol and its analogues are superior to other free radical scavengers in removing oxygen free radicals, emphasizing their core role in removing reactive oxygen (ROS) and inhibiting membrane lipid peroxidation. According to a 2022 study, VKH2, the fully reduced form of vitamin K, can act as a reactive oxygen (ROS) remover and prevent ferroptosis (Mishima et al., 2022). VKH2 not only has a strong ability to remove free radicals, but also has antioxidant properties. It can also inhibit the production of lipid peroxidation, thus avoiding ischemic-reperfusion damage associated with ferroptosis (Ward and DeNicola, 2022).

### 9.4 Inhibitors of the system XC-GSH-GPX4 pathway

The axis of the XC-GSH-GPX4 is the core mechanism for regulating ferroptosis. It is worth noting that GPX4, as a selenium protein, depends on the synthesis of selenium cysteine (Zheng and Conrad, 2020). Selenium (Se) is an essential trace element in the human body. It has significant antioxidant ability and can regulate brain function, thus reducing oxidative stress damage in the brain (Whanger, 2001). Recent studies have shown that exogenous supplementation of selenium (Se) can increase the production of lipid peroxidase GPX4 and inhibit ferroptosis (Panda et al., 2023). Selenium determines the generation of GPX by activating nuclear activation factors, namely, specific protein 1 (Sp1) and activation protein 2γ (AP-2γ) (Alim et al., 2019). Moreover, a different investigation revealed that the chlorinated benzamide derivative 2-amino-5-chloro-N,3-dimethylbenzamide halts iron-dependent cell death by shielding GPX4 from breakdown (Wu et al., 2019). Furthermore, DKK-1 teams up with the ovarian-tumor ubiquitin binder OTUB1 to lock SLC7A11 into an active, long-lived state, thereby hampering ferroptosis (Wu et al., 2022; Liu et al., 2019).

All agents listed above represent the therapeutically viable ferroptosis blockers uncovered to date. Nevertheless, the precise modes of operation and prospective clinical utilities of these molecules demand deeper investigation. Clarifying the link between the mechanisms and features of the effects of these substances (especially multi-target compounds), while investigating the potential for combination therapies and formulating more targeted stimulators or repressors, will significantly influence their clinical application.

## 10 Conclusion and perspectives

AD was discovered over a century ago, yet the exact disease-causing process is still not well understood, even though amyloid-beta deposits and neurofibrillary tangles in the brain are the most common signs of Alzheimer’s disease pathology. The specific causes of nerve cell loss and brain tissue breakdown in Alzheimer’s disease remain unknown, and the underlying process of the illness is intricate. At present, there are no substantial treatment options that can modify the course of the disease. Ferroptosis is a recently identified, a programmed mode of cellular demise, clearly separate from apoptotic, necrotic, and autophagic routes, is chiefly governed by iron homeostasis, lipid peroxide accumulation, and glutathione-dependent biochemical processes. A large number of preclinical studies have shown that treating Alzheimer’s disease against the ferroptosis mechanism is an efficient method, which can significantly improve the success rate of treatment and reduce the burden on patients with Alzheimer’s disease. In addition, ferroptosis inhibitors, such as iron chelators, reactive oxygen scavengers and antioxidant stressors, although their effects are limited, they can still inhibit the neurodegenerative cascading response of Alzheimer’s disease-related injuries to a certain extent.

Clinical trials for AD exhibit high rates of attrition, with physiological barriers to drug delivery and off-target toxicity representing two significant limitations. The BBB constitutes the primary challenge. The endothelial cells of the BBB tightly regulate cerebral entry of substances through junctional complexes, efflux transporters such as P-glycoprotein, and limited endocytosis. Many compounds that demonstrate potential in preclinical models—including those targeting ferroptosis or related pathways—often fail to achieve therapeutic brain concentrations due to suboptimal molecular size, charge, or lipophilicity. Furthermore, even BBB-penetrant drugs may exhibit heterogeneous distribution, limiting efficacy in key affected brain regions.

Systemic toxicity, meanwhile, frequently contributes to trial termination or failure. Small-molecule drugs designed to modulate central nervous system pathways such as iron metabolism, antioxidant defense, or lipid peroxidation often affect analogous targets in the systemic circulation, disrupting normal peripheral organ function. For instance, systemic iron chelation may induce anemia or electrolyte disturbances, while potent lipophilic antioxidants can perturb hepatic metabolism or elicit unintended immunomodulatory effects. These off-target effects create a narrow therapeutic window wherein the effective dose approaches the toxic dose, thereby complicating the maintenance of safe tolerability in chronic human studies.

Given the complexity of AD, a comprehensive prevention and control strategy may be more effective than single-target intervention. Clinical studies have shown that traditional mind-body interventions such as Tai Chi can improve cognitive function, reduce neuroinflammation and enhance neural plasticity (Teng et al., 2024). Combined with targeted therapy of ferroptosis inhibitors, this multimodal approach may provide more comprehensive protection for AD patients. Future research should explore the synergistic effects of drugs and lifestyle interventions.

In a word, this review explains ferroptosis and its common molecular mechanisms, clarifies the role of ferroptosis in Alzheimer’s disease, and summarizes the drugs for the treatment of AD for the mechanism of ferroptosis. However, the research on ferroptosis is still in the early stage, and a large number of experimental studies are still needed to further increase our awareness and understanding of it. It is expected that future research will further reveal more functions and roles in the development of ferroptosis and disease. Based on the unique mechanism of action of ferroptosis, we hope to research a new, safer and more effective inhibitor for ferroptosis targets in the future, and realize its successful clinical transformation and application.
